# Surgical treatment of recurrent spinal phosphaturic mesenchymal tumor-induced osteomalacia: A case report

**DOI:** 10.1097/MD.0000000000018603

**Published:** 2020-01-24

**Authors:** Shuzhong Liu, Xi Zhou, An Song, Zhen Huo, Yipeng Wang, Yong Liu

**Affiliations:** a Department of Orthopaedic Surgery, Peking Union Medical College Hospital, Peking Union Medical College and Chinese Academy of Medical Sciences; b Department of Endocrinology, Key Laboratory of Endocrinology, National Health and Family Planning Commission; c Department of Pathology, Peking Union Medical College Hospital, Chinese Academy of Medical Science and Peking Union Medical College, Beijing, China.

**Keywords:** tumor-induced osteomalacia, spine, hypophosphatemia, phosphaturic mesenchymal tumor, osteoplasty, tumor resection, recurrence

## Abstract

**Rationale:**

Tumor-induced osteomalacia (TIO) is a highly unusual disease with enormous difficulties in clinical diagnosis and curative managements. The objective of this study is to report a very rare case who underwent surgical treatment of recurrent spinal phosphaturic mesenchymal tumor. The management of these unique cases has yet to be further elucidated.

**Patient concerns:**

A 52-year-old man presented with a 3-year history of back pain and 1-year history of continuous and progressive systemic bone pain. The patient, who had been diagnosed of TIO for 3 years, received surgical treatment of extended resection of spinal phosphaturic mesenchymal tumor at L5. Somatostatin receptor tomography revealed the expression of somatostatin in the spine increased significantly, with high suspicion of recurrent phosphaturic mesenchymal tumor.

**Diagnosis:**

Magnetic resonance imaging of spine and positron emission tomography-computed tomography showed the mass in L5, which was highly indicative of the recurrent pathogenic tumor. Postoperative pathology confirmed the diagnosis of phosphaturic mesenchymal tumor in the spinal region.

**Interventions:**

The patient underwent posterior L5 tumor resection, bone cement reconstruction, L4-S1 spinal canal decompression, and L3-S2 internal fixation.

**Outcomes:**

The patient's symptoms improved significantly after the surgery, and we noticed that his hypophosphatemia was successfully corrected after the 2nd operation. Follow-up at 1 month after surgery revealed no recurrence, and the serum phosphorus level of the patient turned to be normal postoperatively. There were no complications associated with the operation during the follow-up period.

**Lessons:**

Taken together, the lesion's clinical features, imaging results, and pathologic characteristics are unique. Combined efforts of specialists from orthopedics, endocrinology, nuclear medicine, radiology, pathology, and medical oncology led to the successful diagnosis and management of this patient. TIO, although rare, should be part of the differential diagnosis when the patient has a history of hypophosphatemia and systemic multiple bone pain. We recommend surgical treatment of the phosphaturic mesenchymal tumor in the spinal region. Osteoplasty by bone cement may be a treatment option for patients with TIO who cannot undergo appropriate surgery or decline open surgery.

## 1 Introduction

Tumor-induced osteomalacia (TIO), also known as oncogenic osteomalacia, is an extremely rare paraneoplastic syndrome characterized by hyperphosphaturia, hypophosphatemia, and elevated alkaline phosphatase levels.^[1–3]^ Clinical manifestations of this condition usually include bone pain, pathologic fracture, and musculoskeletal weakness, which are closely related to a reduction in renal phosphate consumption and bone mineralization.^[2,4,5]^ TIO was 1st described by McCance in 1947. To date, <500 cases of TIO have been reported in the literature.^[1–3]^ Therefore, most clinicians have little knowledge of this rare disease and lack clinical experience in diagnosing and treating TIO. This report describes the clinical manifestations, imaging features, diagnosis, and treatment of a recurrent phosphaturic mesenchymal tumor (PMT) of the spine. We also review cases of TIO published in the English literature, and focus on the clinical manifestations and treatment strategies of this rare disorder.

## 2 Case report

**Figure 1:**
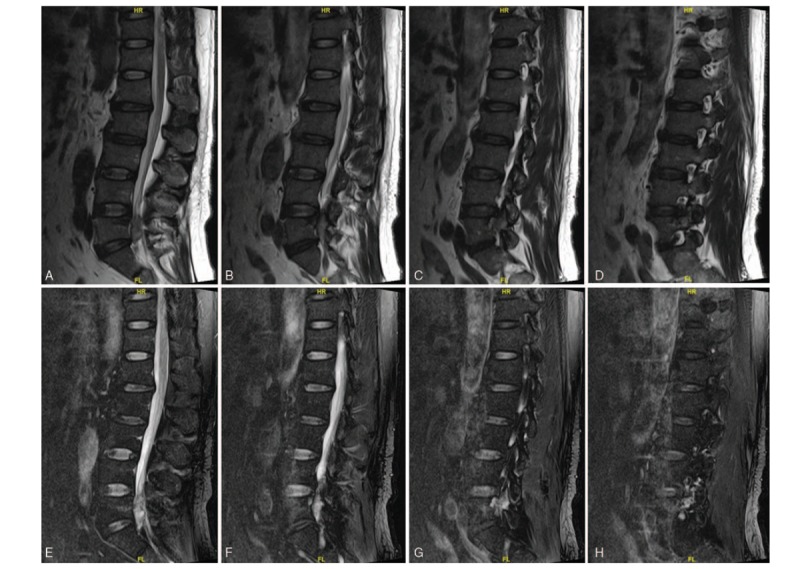
(A–H) Sagittal plane of the magnetic resonance imaging scan revealed the mass in the spine, which was highly indicative of the pathogenic tumor.

**Figure 2:**
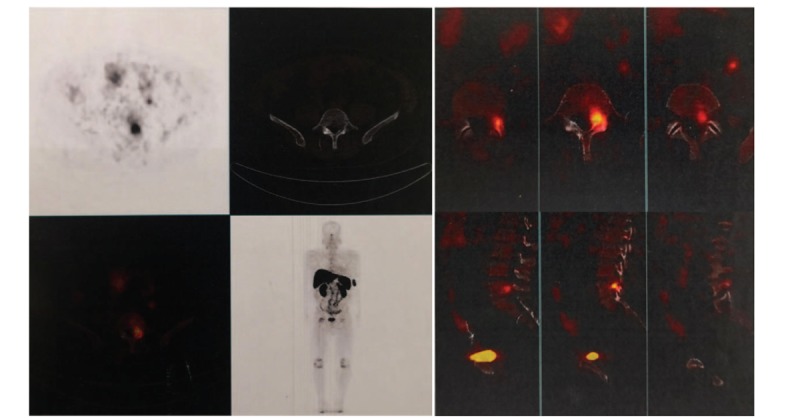
Positron emission tomography-computed tomography revealed the pathogenic tumor at L5.

**Figure 3:**
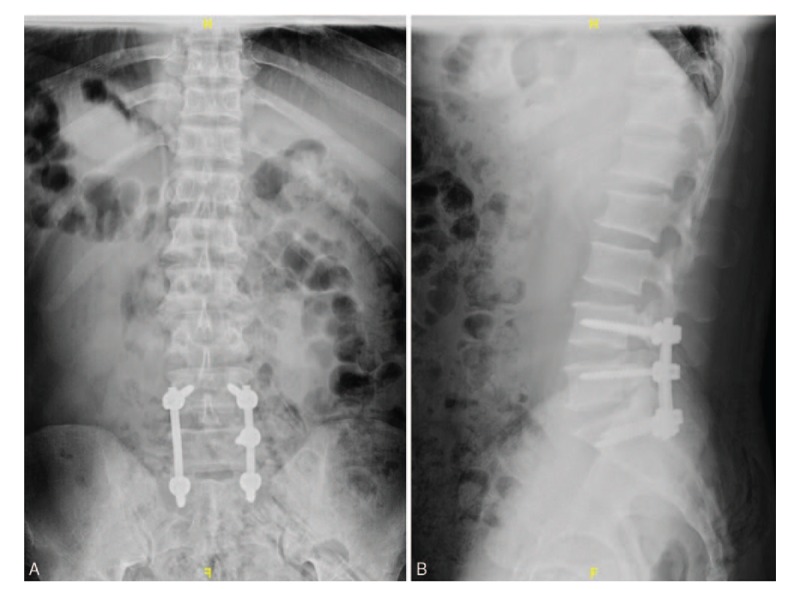
(A, B) Posteroanterior and lateral X-ray images of the lumbar spine obtained after the 1st operation.

In February 2019, a 52-year-old man presented to our hospital with a 6-month history of increasing systemic multiple bone pain and muscle weakness. In history of present illness, the patient stated he had been experiencing a progressive systemic bone pain and general weakness for 6 months, and continuous and progressive hypophosphatemia for approximately 1 year. The pain in his chest and back can reach 8 to 9 points using visual analog scale (VAS) and cannot be alleviated with rest and hot compresses. It is extremely difficult for the patient to turn over, bend down, and change postures. The patient denied experiencing any other constitutional symptoms and the history of adefovir dipivoxil and any other drugs in the past years. Upon further questioning, he recalled a 3-year history of spinal phosphaturic mesenchymal TIO which was confirmed in our institution in March 2016. Magnetic resonance imaging (MRI) of spine showed the pathogenic tumor was in the left posterior part of the spinal canal at the level of L5, and the dural sac was compressed (Fig. 1A–H). Moreover, positron emission tomography-computed tomography (PET/CT) revealed increased uptake in the left side of L5 with high suspicion of PMT (Fig. 2). The patient underwent posterior lumbar spinal decompression, resection of pathogenic tumors at L5, and internal fixation from L4 to S1 (Fig. 3 A and B). Pathologic findings were consistent with PMT with multinucleated giant cell reaction and abnormal calcium deposition, with 1% Ki-67 positive nuclei. The blood inorganic phosphorus increased from 0.49 to 1.04 mmol/L after the 1st operation. The patient was treated with calcitriol (0.25 UG, twice daily), calcium carbonate D3 (600 mg, twice daily), and neutral phosphorus oral solution supplementation (50 mL per time, 5 times a day), and the blood phosphorus level was monitored regularly. In 2017, the patient gradually developed the above symptoms after withdrawal of drugs. Before admission, the patient developed hypophosphatemia for approximately 1 year. The lowest blood phosphorus level was 0.44 mmol/L. No pertinent family history was identified, including hypertension and cancer.

On physical examination, the patient showed pressure pain and percussion pain in his whole spine, chest and pelvis, normal sensation to pin-prick and fine-touch of his bilateral lower limbs and the patient exhibited a 5/5 strength in his bilateral lower limbs. Deep tendon reflexes revealed normal for knee jerk and Achilles tendon reflexes bilaterally. Ataxia, cranial nerves, mini mental, and the rest of the neurologic examination showed no abnormalities. Preoperative hemodynamic and cardiovascular assessments included electrocardiogram, echocardiogram, and chest X-ray. Preoperative laboratory assessment was conducted, including routine laboratory tests (electrolytes, liver and kidney function tests, complete blood count), tumor markers, and endocrinologic evaluation. The results of the laboratory studies were almost within normal range, except that the hypophosphatemia (0.53 mmol/L; normal: 0.81–1.45 mmol/L), reduced alkaline phosphatase (131 IU/L; normal: 45–125 IU/L), and elevated serum neuron-specific enolase (21.4 ng/mL; normal: 0–16.3 ng/mL). Osteomalacia was initially diagnosed, with high suspicion of recurrent TIO.

**Figure 4:**
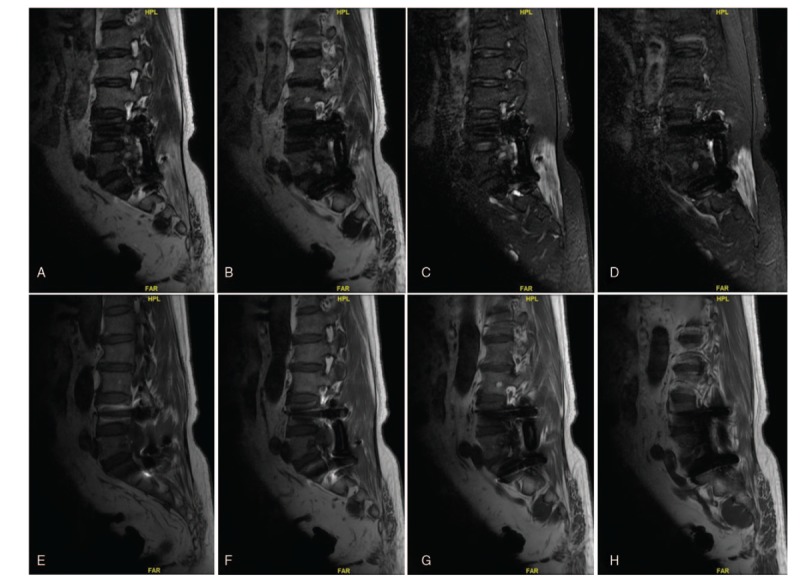
(A–H) Sagittal plane of the magnetic resonance imaging scan revealed the tumor in the spine, with high suspicion of the recurrent pathogenic tumor.

**Figure 5:**
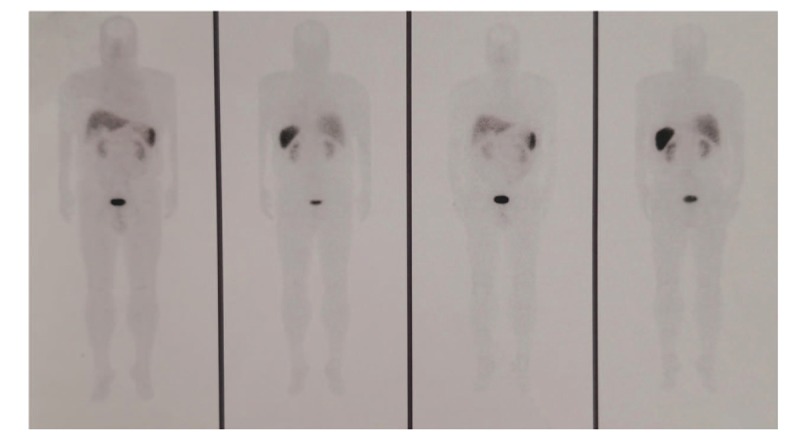
Somatostatin receptor tomography revealed the expression of somatostatin in the spine increased significantly, with high suspicion of recurrent phosphaturic mesenchymal tumor.

**Figure 6:**
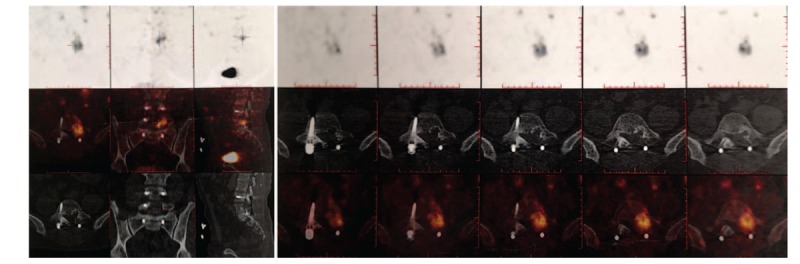
Positron emission tomography-computed tomography revealed the recurrent pathogenic tumor at L5.

The MRI showed the mass at L5, which was highly indicative of the locally recurrent pathogenic tumor (Fig. 4A–H). MRI revealed abnormal signal of the spine in keeping with infiltration of the pathogenic tumor, and mild spinal cord compression secondary to the epidural componant of the L5 mass, with increased metastatic marrow infiltration of the left L5 vertebral and paravertebral regions (Fig. 4A–H). Somatostatin receptor imaging revealed the expression of somatostatin at L5 increased slightly, with high suspicion of pathogenic tumor (Fig. 5). Furthermore, PET/CT scan revealed the high-intake lesion in the spine (Fig. 6). Tumor infiltrated through the L5 vertebral bodies into the left pedicles and posterior elements. Extraosseous spread into the left lateral aspect of the epidural space extending posteriorly, resulting spinal cord compression. Based on these findings, locally recurrent PMT at L5 was considered.

**Figure 7:**
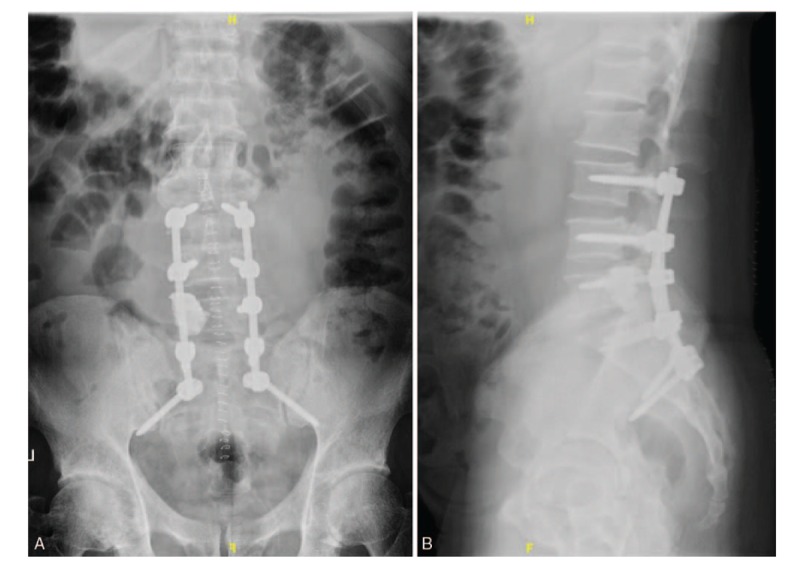
(A, B) Posteroanterior and lateral X-ray images of the lumbar spine obtained after the 2nd operation.

**Figure 8:**
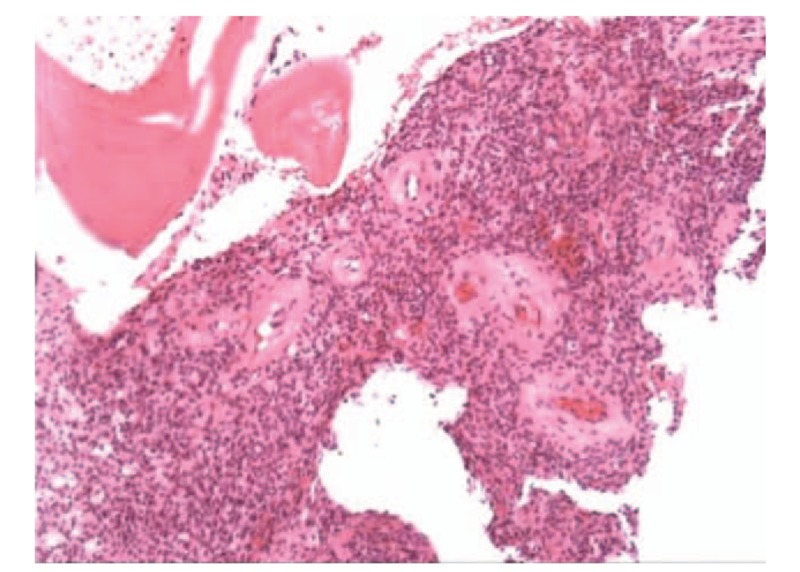
Pathologic histology of tumor specimens was consistent with the diagnosis of phosphaturic mesenchymal tumor.

In brief, posterior complete tumor resection, bone cement reconstruction, spinal canal decompression from L4-S1, and L3-S2 internal fixation were performed according to the original surgical plan. After successful anesthesia, the patient was placed in a prone position for dorsal access to the lumbar spine. For the posterior approach, the paraspinal muscles were detached gently on each side after a midline longitudinal incision was made over the spinous processes. The pedicle entry points were exposed by step-by-step bilateral dissection. At 1st, the previously fixed Sino rods were removed, and the routine screw preparation for bilateral L3 and S2 pedicles were done. The C-arm fluoroscopy showed the location of screw paths is accurate and satisfactory, thus Sino pedicle screws were inserted according to the designed paths. Subsequently, the remaining laminae of L4 and S1 were removed, and scar tissue around the dura mater at L5 level was removed to expose the dural sac and bilateral nerve roots. The suspicious pathogenic tumor tissues were found in the left side of the L5 horizontal spinal canal and the left pedicle of L5, which were completely excised and sent for pathologic examination. During the operation, the examination showed that there was no tension in the dural sac and nerve roots, and the dural sac pulsed with satisfactory decompression. Then the bone cement for reconstruction was prepared, and the bone cement of dough phase was filled in the bone defect after resection of L5 lesions. Then fixation using a screw-rod system was employed. Visual inspection using the intraoperative fluoroscopy showed optimal position of all pedicle screws and bone cement. The incision was closed. Intraoperative blood loss was approximately 900 mL, thus we used no erythrocyte and plasma. Postoperatively, the patient was referred to the regular ward. An X-ray after the surgery confirmed the correct positioning of the implants and bone cement, and no signs of displacement of the screws and rods (Fig. 7A and B). Histopathologic examination was performed, and the diagnosis of PMT was made according to the criteria (Fig. 8). The postoperative pathology together with symptoms and examinations were reported to be consistent with PMT.

**Figure 9:**
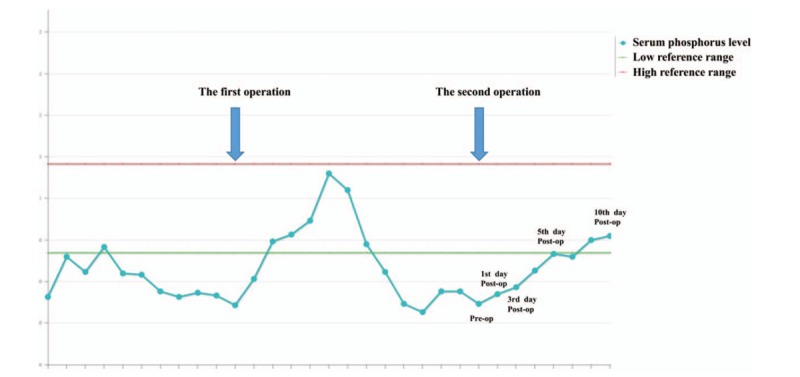
Serum phosphorus levels significantly elevated to the normal range after the 2nd operation.

One week after the operation, the VAS score of his systemic pain improved to 1 to 2 points compared to the preoperative status, 8 to 9 points. Serum phosphorus levels significantly elevated to the normal range (Fig. 9). On physical examination, the patient exhibited a 5/5 strength in the lower extremities. As the patient did not develop severe complications, he was discharged and was monitored as an outpatient. At the 1-month follow-up visit, he had nearly full complete remission and reported no new symptoms.

## 3 Discussion

Osteomalacia is a type of metabolic bone disease, which is characterized by the damage of the mineralization process of the bone-like matrix in mature bone.^[1–3]^ TIO is the most rare type of osteomalacia in the clinic. TIO is commonly located in the craniofacial regions and limbs, and patients who suffer from TIO may have long-term hypophosphatemia.^[4,5]^ We experienced a rare case of a recurrent PMT in the spinal region. TIO can lead to a variety of complications and seriously affect the quality of life.^[1–3,6–10]^ Typically, TIO affects adults, with an even distribution of sex.^[1–3]^ Diffuse bone pain caused by poor bone mineralization is the most common symptom in patients with TIO.^[1–3,11,12]^ Because not many cases of TIO have been previously reported, most clinicians have little knowledge of this disease. Additionally, the clinical process of TIO is often slow, and the tumor size is usually small. Because of the lack of specificity in clinical manifestations, TIO is difficult to locate and diagnose, and it is easy to miss its diagnosis or have misdiagnosis. If the treatment for TIO is not appropriate or timely, severe osteomalacia may lead to progressive and fatal consequences in some cases.^[13–17]^

Notably, one of the most challenging aspects in diagnosing and treating TIO is to find the pathogenic tumor. The feature of PMT delays recognition, diagnosis, and treatment of TIO to some extent. In our case, identifying the tumor and making a final diagnosis took 1 year. According to our review of the literature in PubMed, the average time from the onset of symptoms to the final diagnosis of TIO is usually longer than 2.5 years.^[1–3,8–11,17,18]^ The tumor is often small and slow growing, and located in a specific or atypical site. Therefore, the potential tumor cannot be easily found, and the final treatment is usually delayed for an average of 5 years.^[8–11,17,18]^ Typical laboratory test results include high urinary phosphate, high alkaline phosphatase, and low serum 1,25-dihydroxy vitamin D levels.^[1–3,12,13]^ Hypophosphatemia is secondary to inhibition of renal phosphorus reabsorption, and vitamin D synthesis defects prevent a compensatory increase in calcitriol stimulated by hypophosphatemia.^[12–14,19]^ Additionally, fibroblast growth factor-23 (FGF-23) plays an important role in the pathophysiologic mechanism of TIO. FGF-23 is highly expressed in pathogenic tumors compared with normal tissue.^[1–3,15–20]^

In imaging, TIO shows cortical thickness, osteoporosis, and pathologic fracture.^[1–5,21,22]^ Unfortunately, in most cases, there is difficulty in traditional imaging technology to detect PMT. The classic detection method of TIO is ^99^mTc octreotide imaging, which is a scanning technology for detecting somatostatin receptor expression.^[21–23]^ However, because lymphocytes can also express octreotide receptors, nonspecific uptake may lead to false-positive scans of inflammatory tissue, fractures, or other tumors. Similarly, a negative scan of octreotide cannot completely exclude the diagnosis of TIO. This situation emphasizes the necessity of further detection and differential diagnosis of the tumor by PET/CT or whole-body MRI (WB-MRI). However, because of the tumor's size and occult nature in TIO, despite the use of PET/CT, there may be no increase in uptake.^[14–17,23,24]^ WB-MRI is radiation free and provides excellent contrast resolution for bone, soft tissue, and subcutaneous areas.^[22–24]^

The ideal treatment for TIO is complete tumor resection of the pathogenic tumor, and this corrects the biochemical abnormalities and remineralizes the bone substance in most cases with TIOs.^[1–3,25,26]^ Due to the complexity of spinal anatomy, PMTs are often difficult to be completely removed. Thus, the partial or subtotal resection might also lead to persistent serum abnormalities remained or tumor recurrence. Rigid internal fixation and effective brace should be insured since osteomalacia may reduce bone resistance and increase the risk of bone nonunion or lead to fracture.^[1–6,25–27]^ Extent of surgical resection is reported to be correlated with overall survival benefit, and en bloc tumor resection with spinal stabilization is reported to be an option of surgical treatment.^[28–30]^ However, complete resection may also easily lead to many complications, such as spinal instability, decreased spinal flexibility, neurologic symptoms, neurologic injury, and so on.^[28–30]^ Osteoplasty by cement augmentation may also be a treatment option for patients with PMTs in the spine, who cannot undergo appropriate surgery or decline open surgery.^[28–30]^ However, we need to fully recognize the potential risk of complications in bone cement applications. The safety of this approach still needs to be confirmed in further studies with larger sample sizes and longer follow-up periods. One postoperative complication was cement leakage into the canal and subsequent spinal cord compression.^[27–30]^ The other notable complication is local recurrence after surgery. Under these circumstances, surgical extent, cement volume, and postoperative complications are critical factors that need further investigation.^[28–32]^ With regard to TIO without accurate location, the combination of vitamin D, phosphorus supplementation, and calcitriol can be used to replace progressive renal phosphorus loss, promote renal production of 1,25-dihydroxy vitamin D, and enhance renal phosphorus reabsorption.^[27–32]^ However, medical therapy cannot maintain long-term efficacy and potential complications should also be noted, such as hyperparathyroidism, hypercalcemia, and kidney stone formation.^[17,18]^

## 4 Conclusion

The TIO caused by a PMT is rare, and only a few cases in the spinal region have been published. However, this condition may cause serious symptoms and various complications. In summary, this is the 1st reported case of osteomalacia caused by a recurrent PMT in the spinal region. Although osteomalacia caused by a tumor is uncommon, it should be a considered when a patient has a history of hypophosphatemia and systemic bone pain and weakness in the whole body. We suggest complete resection for treating PMTs. For patients with a spinal PMT who cannot have appropriate surgery or who refuse open surgery, bone cement augmentation may be a proper treatment option. This is a safe and effective procedure for relieving pain of patients with spinal TIO and to help localization of the tumor.

### 4.1 Ethic statement

Written informed consent was obtained from the patient for publication of this article, a copy of which is available for review from the editors of medicine. Because this article does not involve any human or animal trials, it did not require institutional ethical review and approval.

## Acknowledgment

The authors thank our colleagues at the Department of Orthopaedic Surgery, Peking Union Medical College Hospital, Chinese Academy of Medical Sciences and Peking Union Medical College.

## Author contributions

**Conceptualization:** Shuzhong Liu, Xi Zhou, An Song, Yipeng Wang, Yong Liu.

**Funding acquisition:** Shuzhong Liu, Yipeng Wang, Yong Liu.

**Investigation:** Shuzhong Liu, Xi Zhou, Yong Liu.

**Project administration:** Shuzhong Liu, Yong Liu.

**Resources:** Shuzhong Liu, Xi Zhou, Zhen Huo, Yong Liu.

**Supervision:** Yipeng Wang, Yong Liu.

**Writing – original draft:** Shuzhong Liu, Xi Zhou, An Song.

**Writing – review & editing:** Shuzhong Liu, Yipeng Wang, Yong Liu.
